# Ruxolitinib-Associated Phaeohyphomycosis: A Case Report

**DOI:** 10.7759/cureus.19335

**Published:** 2021-11-07

**Authors:** Rory Marples, Milton Micallef, Claudia Whyte

**Affiliations:** 1 Department of Surgery, Prince of Wales Hospital, Sydney, AUS; 2 Department of Infectious Diseases, Prince of Wales Hospital, Sydney, AUS

**Keywords:** myelodysplasia, fungal infection, janus kinase inhibitor, ruxolitinib, cellulitis, infectious disease

## Abstract

We present the findings of a rare fungal infection phaeohyphomycosis caused by *Pleurostoma richardsiae (P. richardsiae) *in an immunocompromised 82-year-old male with myelodysplastic syndrome on treatment with the Janus kinase inhibitor ruxolitinib. Hematologists, infectious disease physicians, and surgeons should consider a fungal etiology in cases of cellulitis refractory to standard treatments in those patients with underlying immune dysfunction and/or in those receiving therapy with ruxolitinib or similar agents.

## Introduction

*Pleurostoma richardsiae (P. richardsiae)*, formerly known as *Phialophora richardsiae*, is a filamentous, dematiaceous fungus found in soil and vegetation in tropical climates and which rarely causes infection in humans. When it does, the infection usually manifests as a subcutaneous cystic collection of fluid known as phaeohyphomycosis, most commonly the result of trauma and direct seeding into the tissue planes. Cases of immunocompromised patients presenting with phaeohyphomycosis are exceedingly rare. We report a case of *P. richardsiae* from right lower limb subcutaneous collections of an immunocompromised 82-year-old man in Sydney, Australia, who was being treated with ruxolitinib for overlap myelodysplastic syndrome and myeloproliferative disorder.

## Case presentation

**Figure 1:**
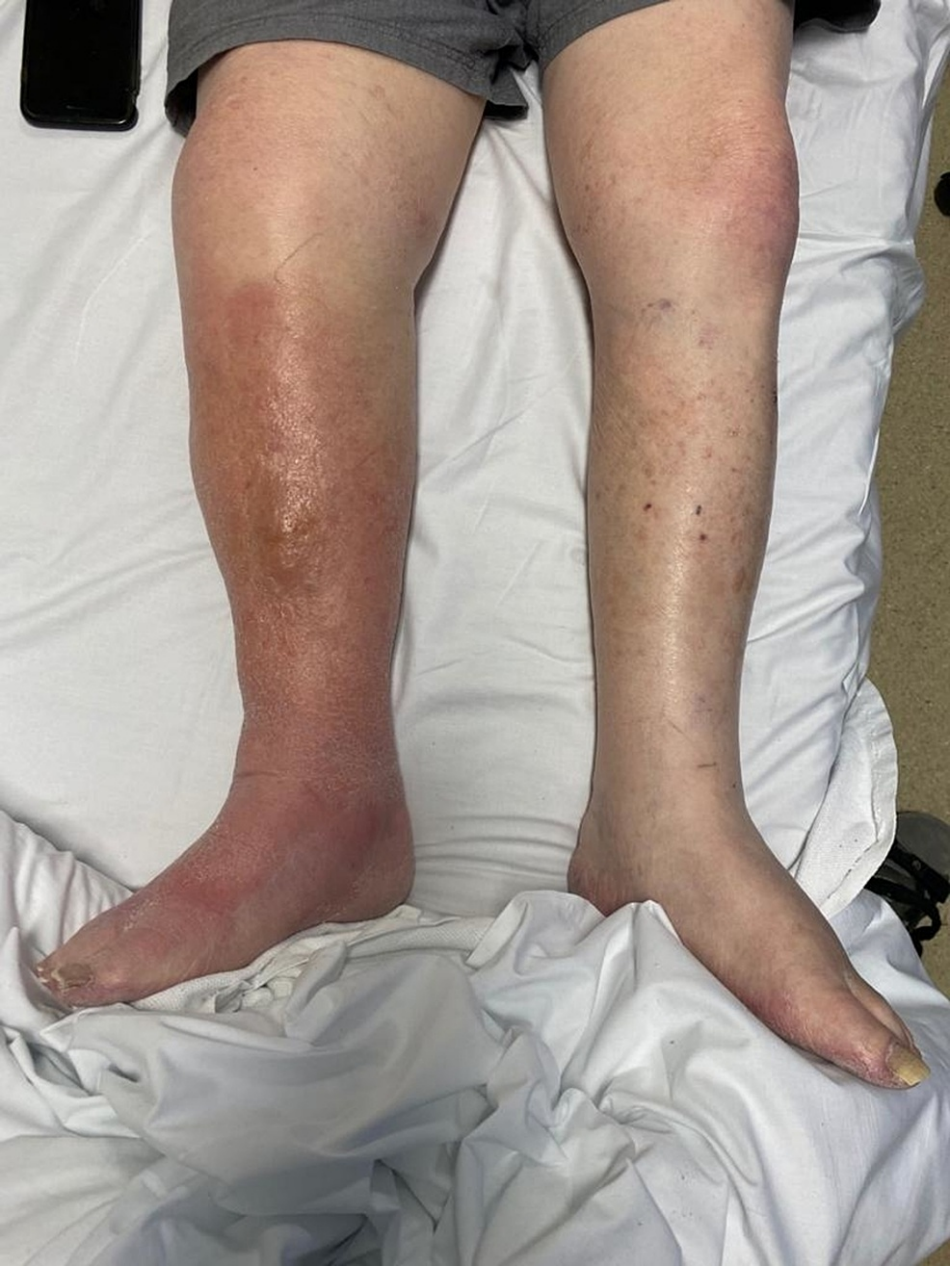
Clinical photograph of patient’s leg prior to surgery.

An 82-year-old man presented to the ED with symptomatic bradycardia, nausea, vomiting, and right lower limb swelling in the context of an acute-on-chronic kidney injury, complicated by a left nephrectomy five years prior, as well as right lower limb cellulitis for which he had recently completed a course of oral clindamycin 450 mg three times daily (TDS). The patient had a background of overlap myelodysplastic syndrome and myeloproliferative disorder for which he had been medicated with ruxolitinib 20 mg every 12 hours (q12h) (a Janus kinase inhibitor) for six months commencing in January 2020, as well as suffering from ischemic heart disease complicated by congestive cardiac failure and atrial fibrillation. As well as bradycardia, he presented with a temperature of 38.1 degrees celsius, oxygen saturations of 93% on room air, and a respiratory rate of 26 breaths per min ute. He was admitted to the hospital under the infectious diseases team for cellulitis and to manage his junctional bradycardia related to his ischemic heart disease, and his acute kidney injury. Despite correction of the bradycardia and acute kidney injury over the course of three days, the cellulitis failed to improve and was refractory to treatment directed both against typical skin organisms (i.e. flucloxacillin 2 g q6h IV) and against methicillin-resistant *Staphylococcus aureus* ([*S. aureus*] of which he was a known carrier; vancomycin IV targeted to trough level of 20 mg/L). It became apparent that there was progression in size of a palpable fluid-filled collection in the pre-tibial area, which it was determined to aspirate for culture on day three of admission (Figure 1).

Within 8 hours, fungal elements were reported on initial microscopy, and within 30 hours were flagged in the blood culture bottle inoculated with fluid from the pre-tibial aspirate. The patient was commenced at this point on liposomal amphotericin B (270 mg [3 mg/kg] q24h). After three days of amphotericin B, which corresponded with a further decline in renal function, therapy was switched to posaconazole 400 mg q12h (as oral suspension).

**Figure 2:**
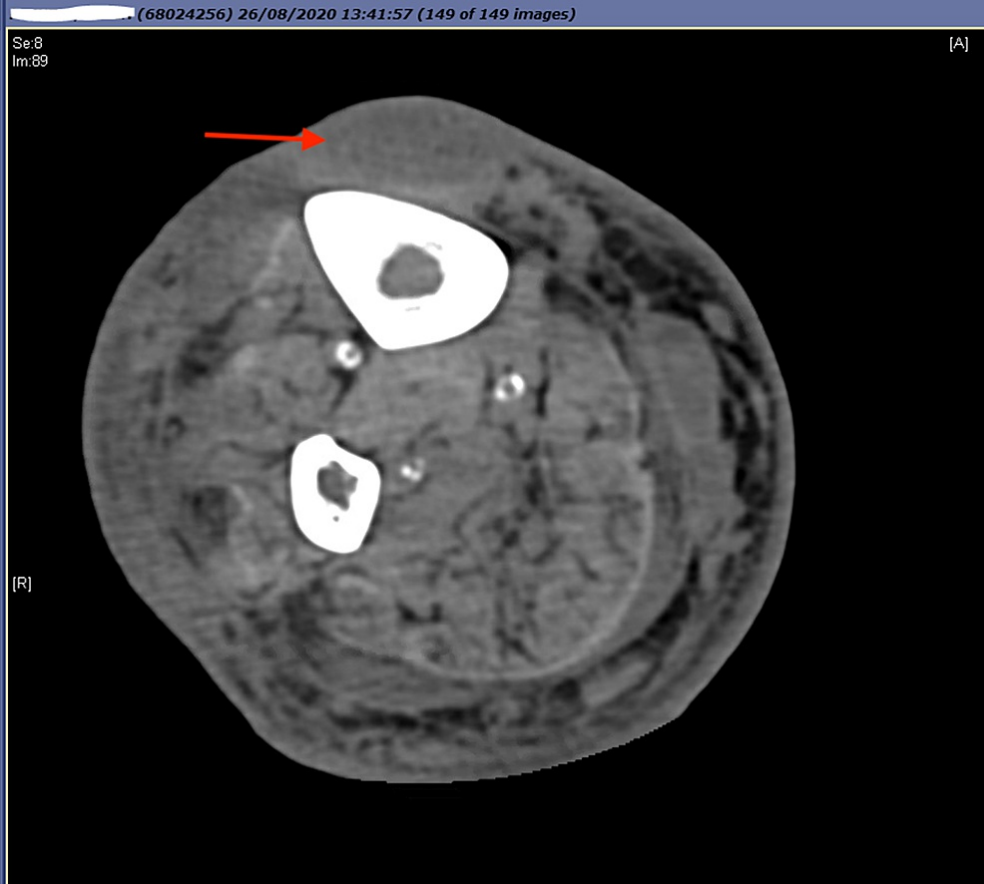
Axial CT scan of the right tibia + fibula demonstrating an anterior tibial collection (red arrow).

A CT scan demonstrated multiple focal subcutaneous collections in the right leg extending from the mid-tibial area to overlying the lateral malleolus, but no evidence of osteomyelitis (Figure 2). A plastic surgery consultation was sought; following consideration, the decision was made for operative management of the collection. Incision and drainage of the collection were undertaken in the operating theatre under a regional nerve block due to high anesthetic risk. Purulent material was drained from the collections, which communicated with each other in the subcutaneous plane. Two Penrose drains were placed intraoperatively to assist with further drainage of the collections. Samples were sent for microscopy, prolonged culture and sensitivities, and tissue pathology.

The same fungal organism was subsequently identified from both the initial aspirate and the operative sample, and thanks to a polymerase chain reaction (PCR) and DNA sequencing of the internal transcribed spacer regions and part of the large subunit (25-28S) rRNA, was identified as *P. richardsiae*, formerly known as *Pleurostomophora richardsiae* and hitherto as *Phialophora richardsiae*. Identification had taken six days, and posaconazole was changed to voriconazole, at 400 mg twice a day (BD) for one day, then 200 mg BD thereafter, based on susceptibility results performed by broth microdilution (see Table 1 of susceptibility results).

**Table 1:** Susceptibility results. MIC: Minimum inhibitory concentration.

Antibiotic	MIC mcg/mL
Amphotericin B	4
Anidulafungin	8
Fluconazole	128
Flucytosine	>64
Itraconazole	1
Micafungin	8
Posaconazole	1
Voriconazole	0.5

The patient was later transferred to another tertiary hospital for ongoing management of his myelofibrosis. Unfortunately, it was revealed on follow-up that the patient subsequently underwent an above-knee amputation of the right leg due to ongoing fungal infection and subsequent re-accumulation of the collections.

## Discussion

*P. richardsiae *is a dematiaceous fungus that rarely causes human disease [1]. Subcutaneous collections of dematiaceous fungi are known as phaeohyphomycoses, and most commonly occur in tropical and subtropical climates [2]. Organisms implicated in phaeohyphomycoses include *Exophiala jeanselmei*, *Phialophora spp*, *Bipolaris spp,* and *Wangiella dermatitidis* [3]. Infections are generally the result of trauma and direct seeding, with increased incidence in males, thought to be due to outdoor work where exposure to soil and vegetation is greater. In this case report, the patient was a retired male, however; he was known to ambulate either barefoot or whilst wearing uncovered shoes.

These fungi are characterized by having melanin in their cell wall, a virulence factor that binds free radicals and reduces phagocytic cell function, making it an ideal nidus for infection, even in immunocompetent patients. There are rare cases of immunocompromised individuals developing phaeohyphomycoses after solid-organ (such as liver or kidney) transplantation [4].

Ruxolitinib, a Janus kinase inhibitor, is an immunotherapeutic drug commonly used to combat myelodysplastic syndromes. In our example, the patient was suffering from overlap myelodysplasia and myeloproliferative disorder and had therefore been treated with ruxolitinib for six months prior to presentation. Unfortunately, this appears to have additionally predisposed him to an invasive fungal infection, and therefore the recalcitrant cellulitis. It is unclear how the fungus had invaded his subcutaneous planes, however, there was evidence of mild epidermal breakdown and hemosiderin deposition secondary to chronic venous insufficiency, as well as edema secondary to fluid overload from cardiac and renal failure. There is a high infection risk associated with myelodysplastic syndromes. A study by Polverelli N et al. of 507 patients with myelofibrosis showed a 15% correlation between ruxolitinib use and severe infections listed above, as well as with *Aspergillus* spp [3]. The patient was aged 82 years and had splenomegaly, both independent risk factors for infections in patients treated with ruxolitinib [4].

Previously, treatment for *P. richardsiae* was surgical amputation of the affected limb [5]. However, further advancements in anti-fungal therapy have yielded strong results, allowing for conservative therapy without the need for amputation. In the literature, most severe infections were likewise treated with voriconazole. Other successful agents used include itraconazole and fluconazole for *P. parasiticum infections *[4]. Unfortunately, due to the extent of the disease, our patient was unable to be managed with voriconazole alone and ultimately required an above-knee amputation when conservative measures failed.

## Conclusions

In conclusion, we present the findings of a rare fungal organism, *P. richardsiae*, phaeohyphomycosis in an immunocompromised 82-year-old male with myelodysplastic syndrome on treatment with the Janus kinase inhibitor, ruxolitinib. Hematologists, infectious disease physicians, and surgeons should consider a fungal etiology in cases of cellulitis refractory to standard treatments in those patients with underlying immune dysfunction and/or in those receiving therapy with ruxolitinib or similar agents.
